# GC-Rich Extracellular DNA Induces Oxidative Stress, Double-Strand DNA Breaks, and DNA Damage Response in Human Adipose-Derived Mesenchymal Stem Cells

**DOI:** 10.1155/2015/782123

**Published:** 2015-07-26

**Authors:** Svetlana Kostyuk, Tatiana Smirnova, Larisa Kameneva, Lev Porokhovnik, Anatolij Speranskij, Elizaveta Ershova, Sergey Stukalov, Vera Izevskaya, Natalia Veiko

**Affiliations:** ^1^Research Centre for Medical Genetics, Russian Academy of Medical Sciences, Mosskvorechie Street 1, Moscow 115478, Russia; ^2^V.A. Nasonova Research Institute of Rheumatology, Russian Academy of Medical Sciences, Russia

## Abstract

*Background*. Cell free DNA (cfDNA) circulates throughout the bloodstream of both healthy people and patients with various diseases. CfDNA is substantially enriched in its GC-content as compared with human genomic DNA. *Principal Findings*. Exposure of haMSCs to GC-DNA induces short-term oxidative stress (determined with H2DCFH-DA) and results in both single- and double-strand DNA breaks (comet assay and *γ*H2AX, foci). As a result in the cells significantly increases the expression of repair genes (*BRCA1* (RT-PCR), PCNA (FACS)) and antiapoptotic genes (*BCL2* (RT-PCR and FACS), *BCL2A1*, *BCL2L1*, BIRC3, and *BIRC2* (RT-PCR)). Under the action of GC-DNA the potential of mitochondria was increased. Here we show that GC-rich extracellular DNA stimulates adipocyte differentiation of human adipose-derived mesenchymal stem cells (haMSCs). Exposure to GC-DNA leads to an increase in the level of RNA*PPARG2* and *LPL* (RT-PCR), in the level of fatty acid binding protein FABP4 (FACS analysis) and in the level of fat (Oil Red O). *Conclusions*. GC-rich fragments in the pool of cfDNA can potentially induce oxidative stress and DNA damage response and affect the direction of mesenchymal stem cells differentiation in human adipose—derived mesenchymal stem cells. Such a response may be one of the causes of obesity or osteoporosis.

## 1. Introduction

In 1940s, it was found that the mammal DNA not only is in cell nuclei, but also circulates in peripheral blood [1] (cell-free DNA, circulating DNA, and plasma/serum DNA). Besides, DNA is always present in the cell culture medium (extracellular DNA (ecDNA)). There are two fundamental hypotheses of the plasma/serum cfDNA origin: formation of the pool of extracellular nucleic acids because of cell death and/or active secretion of cfDNA by living cells [2, 3]. The intense interest in cfDNA is related to a possibility of using it for the purpose of diagnostics of various cancers; for the detection of disturbances of fetal development; and for the assessment of risk of disturbing factors, including ionizing and ultraviolet radiation [4–6].

At the present time, the authors' attention is drawn not only to the use of cfDNA as an early marker of diseases and pathological conditions, but also to studying various potential biological functions of cfDNA [6–8]. In pathological conditions and due to impacts hazardous for the genome, a number of cfDNA parameters, such as plasma concentration and content of various DNA sequences in cfDNA, are changed. As part of cfDNA, GC-rich genomic sequences are often accumulated. In cfDNA, GC-content was found to reach 74.8% (with an average of 53.7%), whereas nuclear GC-content is just 38% [9, 10]. Earlier, we found an increased content of fragments of the transcribed region of ribosomal repeat (rDNA) in cfDNA [11, 12]. It is known that GC-pairs form up to 80% of some regions of rDNA. The cfDNA enrichment with ribosomal repeat several times in comparison with the genome ribosomal repeat content typically occurring during a chronic pathology accompanied by intensified cell death (ischemic heart disease and rheumatic arthritis) [11–13], as well as in consequence of a chronic external damaging influence (ionization radiation) [14].

Mesenchymal stem cells of human adipose tissue (haMSCs) are multipotent progenitor cells that can differentiate into several cell types. The haMSCs respond to an alteration of GC-rich sequence content in ecDNA of the culture medium. The GC-rich DNA molecules were shown to serve as ligands for the toll-like receptor family, namely, TLR9. All the ligands for TLR9 contain CpG motifs. The contents of the ligands for TLR9 in DNA directly depend on the GC-content of the DNA. TLR9 stimulation results in an activation of transcription factor NF-kB and producing TNF*α* and IL6 [15]. It is known that NF-kB activation can result in an arrest of adipogenic differentiation and stimulate osteogenesis [16]. Conversely, it was shown recently that a change of the total ecDNA concentration in the culture medium of haMSC is followed by production of reactive oxygen species (ROS), which are considered as agents significantly enhancing adipogenesis [17]. The matter of the direction of haMSC differentiation after changing the properties of ecDNA in the ambient medium is important in terms of* in vivo* response of stem cells for a pathologic process. Moreover, stem cells are used in therapeutic purposes for the introduction into the patient's body. As a rule, in severe conditions, the concentration and GC-content of cfDNA in haMSC recipient's body are significantly changed in comparison with healthy controls. Thus, the aim of this study was an analysis of the influence of normal and GC-rich ecDNA fragments on the level of ROS, double-strand DNA breaks, DNA damage response, and spontaneous differentiation of haMSCs to adipocytes.

## 2. Materials and Methods

### 2.1. Cell Culture

Mesenchymal stem cells (haHaMSCs) were obtained from adipose tissue of patients subjected to surgical operation. To obtain stromal cells, minced adipose tissue was digested with collagenase as described previously [17]. Immunophenotype and other characteristics of collected cells were described earlier [17]. HaMSCs (2278) were cultivated in a humidified atmosphere with 5% CO_2_ in air at 37°C in AmnioMax C-100 Basal Medium (Gibco), containing AmnioMax Supplement C-100. Before treatments, cells were split no more than four times. Fluorescence-activated cell sorting analysis (FACS) has shown that the cultured HaMSCs did express MHC (major histocompatibility complex) molecules (HLA-ABC+) and adhesion molecules (CD44+, CD54 (low), CD90+, CD106+, CD29+, CD49b (low), and CD105); however, these cells were negative for hematopoietic markers (CD34-, CD45-, and HLA-DR-) and the marker CD117 [17]. In presence of an inducer (kit for adipogenic differentiation, “StemCell Technologies Inc.”), these cells underwent differentiation into adipocytes. HaMSCs were cultivated in the presence of DNA samples in a humidified atmosphere with 5% CO_2_ in air at 37°C. Ethical approval for the use of haMSCs was obtained from the Regional Committees for Medical and Health Research Ethics (approval number 5).

### 2.2. DNA Probes

*GC-DNA*: linearized plasmid DNA (10 197 bp) contains rDNA sequences (5836 bp, 73% GC) cloned into EcoRI site of pBR322 vector 4361 bp (53% GC) in length. Cloned rDNA fragment covers positions from −515 to 5321 of human rDNA according to HSU13369, GeneBank. Linearized vector pBR322 served as a control. All DNA samples were subjected to the same lipopolysaccharide-removing purification procedure that included treatment with Triton X-114 followed by gel-filtration on the HW_85 [15]. Genomic DNA (gDNA, ~38–40% GC) was isolated from haMSCs by the phenol extraction method.

### 2.3. RT-PCR Analysis

Total RNAs were isolated from cultured cells using the Yellow Solve kits (Clonogen, Saint Petersburg, Russia) and treated with DNAse I (Sileks, Moscow, Russia). RNAs were quantified with Quant_iTTM RiboGreen RNA reagent (MoBiTec) on a luminescent spectrometer LS 55 (PerkinElmer, England) and their concentrations normalized to the number of cells used for RNA isolation. The reverse transcription was performed using the MMLV-RT (Sileks, Russia) following the standard protocol. The relative abundance of individual mRNAs was assayed by qRT-PCR using SYBRGreen PCR MasterMix (Applied Biosystems) and a StepOnePlus instrument (Applied Biosystems). The levels of RNA were assayed in at least three independent experiments with CV at 2%. TBP was used as the reference gene after an experimental validation of the stability of its expression in haMSCs using TBP, GAPDH, and AKTB as selection pool. The following primers were ordered in Sintol (Russia):

 
*BRCA1* (F: GGCTATCCTCTCAGAGTGACATTTTA, R: GCTTTATCAGGTTATGTTGCATGGT);
 
*BCL2* (F: TTTGGAAATCCGACCACTAA, R: AAAGAAATGCAAGTGAATGA);
 
*BCL2A1* (Bfl-1/A1) (F: TACAGGCTGGCTCAGGACTAT, R: CGCAACATTTTGTAGCACTCTG)
 
*BCL2L1* (BCL-X) (F: CGACGAGTTTGAACTGCGGTA, R: GGGATGTCAGGTCACTGAATG)
 
*BIRC2* (F: GAATCTGGTTTCAGCTAGTCTGG; R: GGTGGGAGATAATGAATGTGCAA)
 
*BIRC3* (c-IAP1) (F: AAGCTACCTCTCAGCCTACTTT, R: CCACTGTTTTCTGTACCCGGA)
 
*NOX4* (F: TTGGGGCTAGGATTGTGTCTA; R: GAGTGTTCGGCACATGGGTA);
 
*LPL* (F: ACAAGAGAGAACCAGACTCCAA; R: GGTAGTTAAACTCCTCCTCC)
 
*PPARG2* (F: ACCAAAGTGCAATCAAAGTGGA, R: GGCTTATTGTAGAGCTGAGTCT);
 
*TBP* (reference gene) (F: GCC CGA AAC GCCGAA TAT, R: CCG TGG TTC GTG GCT CTC T).

### 2.4. Flow Cytometry

For flow cytometry measurement of Ki-67, PCNA, BCL2, FABP4, and *γ*H2AX, haMSCs were washed in Versene solution and then treated with 0.25% trypsin under control of light microscopy. Cells were transferred to the Eppendorf tube and washed with DMEM medium, then centrifuged, and resuspended in PBS. Cells were fixed in 3% PFA for 10 min at 37°C, washed with PBS, and then permeabilized with 0.1% Triton X-100 (Sigma) in PBS for 15 min at room temperature. PCNA, FABP4, and BCL2 were analyzed using specific antibodies (eBioscience, USA) and FITC goat anti-mouse IgG (US Biological, USA). *γ*H2AX and Ki-67 were analyzed using *γ*H2AX-specific and Ki-67-specific antibodies labeled with FITC (US Biological, USA). To quantify the background fluorescence, we stained a portion of the cells with secondary FITC-conjugated antibodies only. Cells were analyzed using CyFlow Space (Partec, Germany); each experiment was repeated at least three times. Subpopulations of the cells were gated as recommended by the CyFlow software.

Annexin V binding assays: following treatment with DNAs, cells were detached by trypsinization, counted and pelleted (1000 r.p.m. for 5 min). Cell pellets were washed once with PBS and once in Annexin V binding buffer (10 mM HEPES, pH 7.4, 140 mM NaCl, and 2.5 mM CaCl_2_). Cells were treated with Annexin V-FITC at room temperature for 15 min in the dark. Cells were analyzed for fluorescence on CyFlow Space.

### 2.5. Fluorescent Microscopy

Cell images were obtained using the AxioScope A1 microscope (Carl Zeiss). Immunostaining was performed as previously described [16]. The cells were washed by PBS, fixed on glass with 3% formaldehyde solution for 15 min at 4°C. The glasses were washed by cold PBS two times for 5 min and by PBS with 0.1% Triton X-100 one time for 10 min, stained with FITS-*γ*H2AX antibody (US Biological, USA) for 1 h at room temperature in PBS with 0.02% Triton X-100. Afterwards we added for 10 min rhodamine phalloidin at concentration of 0.4 U/mL in PBS with 0.02% Triton X-100. Then we washed the cells three times by PBS and analyzed the same fields of the preparations at the excitation wavelength 520–550 nm (red fluorescence) and at the excitation wavelength 488 nm (green fluorescence). At least 100 fields on each preparation were analyzed. We counted the number of stained cells of four types: (1) without clear spots, (2) without the small and high number of clear spots (but clearly resolvable), (3) with the very high number of signals (confluent stained zones), and (4) with mitotic nuclei. The assessment of unspecific sorption was performed with the use of the FITC-labelled mouse immunoglobulin (US Biological, USA).

### 2.6. ROS Detection Assays

The experiments were performed in slide flasks or in the 96-well plates (Nunclon, Germany). Before treatment, cells were grown to subconfluency. After DNA treatment, the cells were incubated at 37°C. To detect ROS production, cells were treated with 10 *μ*M of H2DCFH-DA (Molecular Probes/Invitrogen, CA, USA). Cells were analyzed by each of the following methods: (1) total fluorescence analysis in the 96-well plate format, *λ*_ex_ = 488 nm, and *λ*_em_ = 528 nm (EnSpire Equipment, Finland); and (2) flow cytometry analysis (Partec, Germany). H2DCFH-DA stained cells were washed in versene solution and then treated with 0.25% Trypsin. The trypsinization level was controlled by light microscopy observation. Cells were transferred to the Eppendorf tube and washed with media 199, then centrifuged, and resuspended in 1x PBS. One hundred thousand cells were analyzed using FACS with FL1 laser; each experiment was repeated at least 3 times.

### 2.7. Adipogenic Differentiation

HaMSCs were seeded into slide flasks, grown to subconfluency (~80%) and exposed to DNA samples at concentrations of 50 ng/mL. In a week the culture medium with DNA samples was refreshed. At day 14, adipogenesis was quantified by fixing the cells with 4% PFA and staining with 0.3% Oil Red O solution (Chroma, Münster, Germany) and with CytoGreen (Invitrogen). Cell images were obtained using the AxioScope A1 microscope (Carl Zeiss). Control (−) DNA samples were not added to the medium; control (+) – cells were cultured in the medium reliably inducing adipogenesis (“StemCell Technologies Inc.”). Differentiation experiments were repeated three times.

### 2.8. Statistics

All reported results were reproduced at least three times as independent biological replicates. The figures show the average data and the standard deviation (SD). The significance of the observed differences was analyzed using nonparametric Mann-Whitney* U* tests. *P* values < 0.05 were considered statistically significant и marked at the figures with (^*^). Data were analyzed with StatPlus2007 Professional software (http://www.analystsoft.com).

## 3. Results

**Figure 1:**
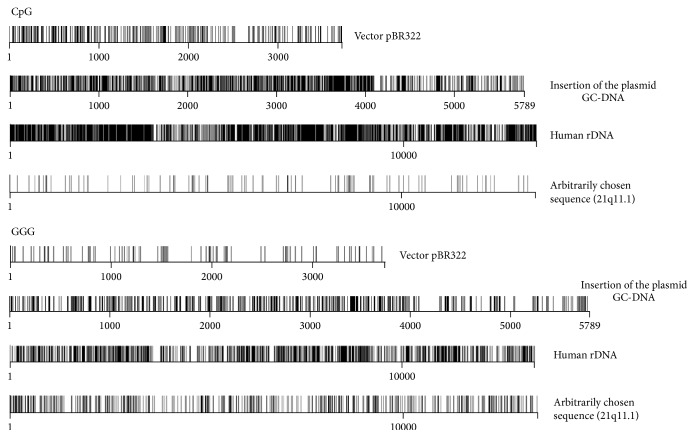
Distribution of CpG-motifs and Gn-motifs (*n* = 3) within the model DNA samples and within rDNA (transcribed region of human ribosomal repeat) that accumulates in circulating DNA of blood plasma. The digits indicate the nucleotide order number, while the vertical bar shows the motif location. For comparison, the figure also presents the distribution of the motifs within a randomly chosen fragment of genomic DNA with a total GC-content of 42%.

This study was performed using subconfluent haMSCs obtained from donor and characterized by CD marker expression. Detailed description of the haMSCs used (line 2278) were presented in our previous work [17]. Untreated MSC culture medium contains endogenous extracellular DNA (ecDNA). Concentrations of endogenous ecDNA in the haMSCs medium averaged to 12 ± 2 ng/mL [15, 17]. In most experiments, a concentration of added DNA probe of 50 ng/mL was used as standard. Two major types of DNA preparations were used: (1) genomic DNA (gDNA) with low GC-content (~38–40%). This DNA was fragmented to shorter fragments using limited hydrolysis with DNAse 1 and (2) DNA with high GC-content. The second type included plasmid-vector pBR322 (53% GC) and GC-DNA plasmid, which contains pBR322 vector and an insertion, a GC-rich fragment of the transcribed region of human ribosomal repeat (rDNA) 5836 bp long (73% GC).  Figure 1 displays the distribution of CpG-motifs, which constitutes the ligands for TLR9, within pBR322 plasmid-vector and within plasmid GC-DNA. The ligands for TLR9 are supposed to be a principal cause of the biological activity of GC-rich DNA [12–15].  Figure 1 also presents the CpG-content within the transcribed region of human ribosomal repeat, which accumulates as part of cfDNA in blood plasma of healthy people and, especially, patients with some chronic pathologies [11–14]. For comparison, the figure also presents the distribution of CpG-motifs within a randomly chosen sequence of genomic DNA of the same length as the transcribed region of human ribosomal repeat. Besides, Figure 1 shows the distribution of Gn-motifs (*n* = 3–5) within the same DNA fragments. Gn-motifs are interesting due to the fact that guanosine contained in them is very oxidation-proned [18]. Consequently, regions of oxidated DNA may occur. Oxidated DNA possesses expressed biological activity and induces oxidative stress in stem cells [17]. The samples of gDNA and GC-DNA contained DNA fragments of approximately equal length: ~11 kb.

### 3.1. An Increase of ecDNA Concentration Elevates the Level of ROS in haMSCs

**Figure 2:**
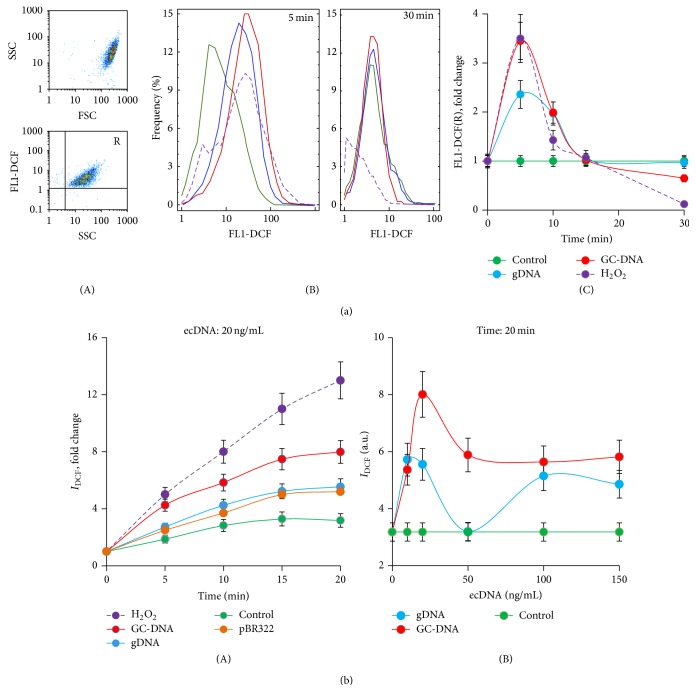
Exposure of haMSCs to different extracellular DNA samples induces short-term oxidative stress. (a). (A) FACS analysis showing the homogeneity of the cells (FSC-SSC diagram) and the stained cellular fractions (SSC-FL1(DCF) diagram). Control haMSC were treated with 10 *μ*M H2DCFH-DA; (B) FACS analysis showing the distribution of haMSC stained with DCF. Cells were pretreated with DNA samples (20 ng/mL) for 5 min and then with 10 *μ*M H2DCFH-DA for 20 min. Gate R encircles the fraction of haMSCs that express ROS. For comparison, the figure shows the data for cells treated with 0.2 mM peroxide; (C) the kinetics of changes in the average intensity of the cells in presence of various DNA samples (20 ng/mL) or 0.2 mM peroxide. (b) (A) the kinetics of changes in *I*_DCF_ (fluorescence reader, *λ*_ex_ = 487 nm and *λ*_em_ = 526 nm). DNAs (20 ng/mL) or 0.2 mM H_2_O_2_ were added after 3 min incubation of MSC with 10 *μ*M H2DCFH-DA. The measurements were carried out 5, 10, 15, and 20 min after addition. Data points were averaged and represented as mean ± SD for eight biological replicates. Background fluorescence values of culture medium in the presence of DNAs or 0.2 mM H_2_O_2_ were taken into account; (B) *I*_DCF_ dependence on the concentration of the added DNAs (incubation time: 20 min). (^*^)  *P* < 0.05.

A change in ROS levels in the cells in response to a change of total ecDNA concentration was studied using FACS analysis (Figure 2(a)). ROS levels were evaluated using H2DCFH-DA (10 *μ*M). The dye was added at 20 min after the incubation of cells with DNAs in the course of a certain period of time, as shown in the figure. The measurements were carried out for gDNA and for GC-DNA (20 ng/mL, Figure 2(a)). Virtually immediately (within the first 5 minutes) after adding DNA probes to the medium, a sharp increase of ROS level in the cells was observed (Figure 2(a)(B)). GC-DNA induced a stronger response than gDNA. ROS level in haMSC after adding GC-DNA was approximately equal to ROS level in the presence of H_2_O_2_ oxidant at 0.2 mM. The effect is observed within the first 15 minutes of incubation of the cells with DNA probes or H_2_O_2_ and further blocked (Figure 2(a)(C)). 30 minutes later, ROS level in the cells treated with GC-DNA or H_2_O_2_ decreases lower than the control one. The signal decline can be associated with either a real reduction of the level of ROS synthesis as a result of antioxidative response or a dye leakage through cell membrane damaged by ROS. The evidence for possibility of DCF leakage is the appearance, with the course of time, of a fraction of unstained cells in a population of haMSCs treated with H_2_O_2_ (Figure 2(a)(A and B)). In case of the impact of DNA probes on haMSCs, no emerging fraction of unstained cells was observed.

The FACS data were independently corroborated by fluorescent reader data. HaMSCs were exposed to 10–150 ng/mL of gDNA, pBR322, and GC-DNA. ROS levels were evaluated using H2DCFH-DA (10 *μ*M) immediately after the addition of DNA probe. In instance, Figure 2(b)(A) presents the data of the impact of DNA probes at 20 ng/mL on haMSCs. All DNA samples stimulated a 2- to 3-fold increase in the levels of ROS produced by haMSCs. GC-DNA in a greater degree stimulated ROS synthesis more than gDNA and pBR322. H_2_O_2_ significantly increased the intensity of general fluorescence, which corroborates the above assumption of dye leakage from the cells, probably, because of membrane breakage. Figure 2(b)(B) shows the dependence of DCF signal on the concentration of added DNA probe. The maximum signal was observed when GC-DNA probe had been added at a concentration of 20 ng/mL. The maximum difference between GC-DNA and gDNA was observed at a probe concentration of 50 ng/mL.

Thus, a sharp increase of ecDNA concentration several times will be accompanied by a fast but short rise of the ROS amount in haMSCs. GC-DNA is a stronger ROS inducer than gDNA and pBR322. The action of GC-DNA is comparable with the effect of 0.2 mM H_2_O_2_, but no damage to cell membrane occurs. High ROS level in the cell can potentially lead to an oxidation of cell's own DNA. One of the well-known consequences of DNA oxidation is an accumulation of single- and double-strand DNA breaks (SSBs and DSBs).

### 3.2. Exposure to gDNA and GC-DNA Induces Strand Breaks in Cell's Own DNA

**Figure 3:**
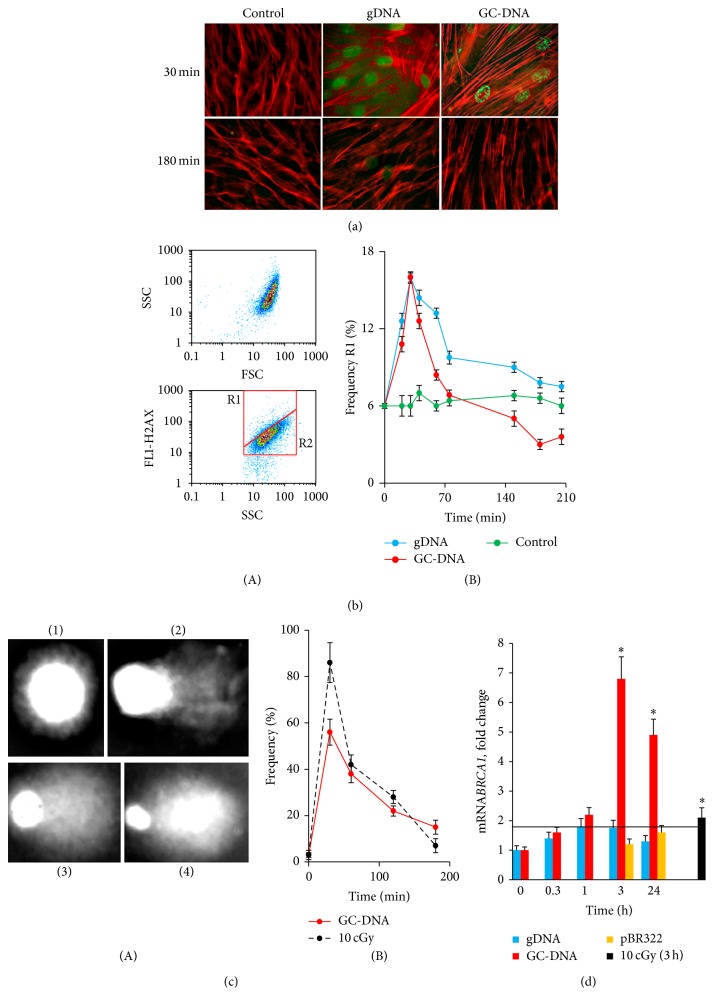
Nuclear DNA damage and repair in haMSCs exposed to DNA samples. (a) *γ*H2AX in cells exposed to gDNA and GC-DNA (50 ng/mL, 30 min and 180 min). Cells were fixed with 3% PFA, treated with 0.1% Triton X-100 and processed for immunofluorescence staining with anti-*γ*H2AX antibody (FITC) (magnification 40x); rhodamine phalloidin was used to label cytoskeletal F-actin. (b) FACS analysis of *γ*-foci. (A) Their main fractions of the cells as evident in gating areas R1 and R2; (B) the kinetics of changes in the relative proportions of cells with *γ*-foci (gate R1). Data points were averaged and represented as mean ± SD for three biological replicates. (^*^)  *P* < 0.05 against control group of cells, nonparametric* U* test. (c) Comet assay in alkaline conditions. (A) Digital photography of the nuclei with varying degree of DNA damage. (B) The kinetics of changes in the proportions of haMSCs with SSB and DSB. HaMSCs were exposed to GC-DNA (50 ng/mL) or to ionizing radiation (10 cGy). (d) The ratio of the levels of mRNA*BRCA1 *in cells exposed to 50 ng/mL gDNA, pBR322, or GC-DNA or to ionizing radiation (10 cGy). The incubation time is indicated on the histogram. Data points were averaged and represented as mean ± SD for three biological replicates. (^*^)  *P* < 0.05 against control,* U* test.

To quantify DSBs in haMSCs exposed to either gDNA or GC-DNA, we employed a common technique for the visualization of DSBs, immunostaining with antibodies against the histone *γ*H2AX, phosphorylated at serine-139. This form of H2AX is known to rapidly accumulate at DNA loci flanking the DSB site [19]. HaMSCs stained with FITC-conjugated antibodies to Ser-139 phosphorylated histone *γ*H2AX are shown in Figure 3. One of the main consequences of a ROS burst is the process of actin polymerization/depolymerization [20]. For the visualization of the polymeric form of actin (F-actin) along with *γ*H2AX, we used the conjugate of falloidin with rhodamine [21].  Figure 3(a) also shows that the amount of F-actin in relation to the control significantly increases after an exposure to gDNA or GC-DNA, with the staining intensity of this protein per cell being more than that of the control cells. The actin stress-fiber formation generally takes place after a ROS burst [22].

**Figure 4:**
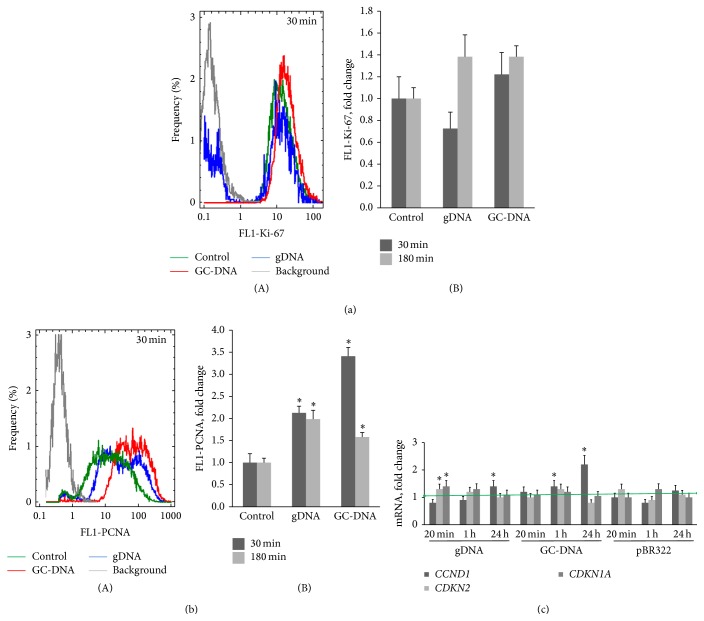
Changes in the amount of proliferation markers Ki-67 and PCNA in haMSCs exposed to gDNA or GC-DNA at final concentration 50 ng/mL (FACS). (a) (A) fixed cells stained with anti-Ki-67 (FITC) antibodies. Distribution of fluorescence intensities of the cells* exposed to DNAs*. Background fluorescence was quantified using FITC-conjugated secondary antibodies (grey color); (B) the average of the median signal intensity of FL1 (Ki-67) in haMSC. (b) (A) fixed cells stained with anti-PCNA (FITC) antibodies. Distribution of fluorescence intensities of haMSCs* exposed to DNAs*. Background fluorescence was quantified using FITC-conjugated secondary antibodies (grey color). (B) The average of the median signal intensity of FL1 (PCNA). (c) The ratio of the levels of mRNA* CCND1, CDKN2, *and* CDKN1A *in cells exposed to gDNA, pBR322, or GC-DNA. The incubation time is indicated on the histogram. Data points were averaged and represented as mean ± SD for three biological replicates. (^*^)  *P* < 0.05 against control, nonparametric* U* test.

In the control samples, 12 ± 3% of the cells are stained with FITC-conjugated antibodies to Ser-139 phosphorylated histone *γ*H2AX. After any kind of DNA is added to the medium, the fraction of cells with *γ*H2AX expression increases (Figure 4(a)) as early as 30 minutes later, but, 3 hours later, it decreases. The decrease is more prominent for GC-DNA. Thus, elevation of ecDNA concentration leads to a short-time increase of the number of cells with clear gamma focuses that indicates an increase of the number of cells with double-strand DNA breaks, the portion of which, in case of gDNA and GC-DNA reaches, respectively, 15% and 19% of the whole pool of haMSCs.

Using FACS, two gated areas, R1 and R2, were studied (Figure 3(b)(A)). Cells within gate R1 have largest FL1 (*γ*H2AX); this is interpreted as multiple DSBs. Gate R2 contains cells with low level of *γ*H2AX and with no DSBs. In the control, R1 fraction makes on the average 6 ± 1% of the whole population. This fraction includes cells with elevated amount of *γ*H2AX marker. The quantity of these cells increases more than 2 times as early as 20 minutes after a rise of concentration of any DNA in the medium, while it begins to decrease 1.5 hours after adding the DNA probe (Figure 3(b)(B)). In the presence of GC-DNA, the decrease is more prominent, and, 2.5 hours after adding GC-DNA to the medium, the quantity of R1 fraction cells decreases 2 times in comparison with the control. Thus, while ecDNA concentration increases in the culture medium, the total amount of *γ*H2AX histone temporarily increases in a portion of the cells. The effect can be observed for no longer than 2-3 hours after the DNA is added to the culture medium.

Since some works were published recently, which reported that even clear gamma focuses do not always correspond to DNA breaks [23, 24], we corroborated the gamma focus data using another technique detecting chromatin breaks. This technique called DNA “comet” assay, allows detecting damaged DNA in a certain separate cell. We employed comet assay electrophoresis in alkaline conditions (Figure 3(c)). The technique enables testing totally both single- and double-strand breaks in the nuclear DNA. The experiment was carried out for GC-DNA (Figure 3(c)(A)). As a reference impact, which is with certainty followed by DNA breakage, ionizing radiation was used at a dosage of 10 cGy. The haMSC pool comprises cells of 4 types (Figure 3(c)(A)). Type 1 cells (they make more than 90% of the control cell pool) contain no DNA breaks. Type 2 cells contain small number of DNA breaks. In type 3 cells, DNA is highly fragmented, and type 4 cells are cells undergoing apoptosis (less than 1% of the reference population) with extremely high extent of DNA fragmentation. Figure 3(c)(B) displays data on changes of the total cell fractions with different extent of DNA breakage (types 2–4) occurring after adding GC-DNA to the medium or after exposure to radiation. In case of GC-DNA, within the first 30 minutes of the experiment we observed formation of cells of, predominantly, type 2 containing small number of breaks. In the population of radiation-exposed cells, in 30 minutes after the exposure, most cells were of type 3. Three hours later, the portion of cells containing DNA breaks came to reduce and converged to the reference level.

We analyzed changes in expression levels for mRNA encoding cell DNA repair related protein BRCA1. In three hours after adding GC-DNA to haMSCs, levels of mRNA for BRCA1 showed a 7-fold increase (Figure 3(d)). In case of treatment with gDNA and pBR322, genes also tend to increase the mRNA biosynthesis, up to 1.5–2 times.

Thus, we have established that a rise of ecDNA concentration in the culture medium of haMSCs is accompanied by* de novo* nuclear DNA breaks, probably, owing to a ROS burst. The DNA breaks are known to block the cell cycle. In turn, this can affect the process of differentiation of haMSCs to adipocytes [25]. For this reason, we studied an influence of DNA probes added upon the cell cycle in haMS cells.

### 3.3. Exposure to gDNA and GC-DNA Did Not Affect Significantly the Cell Cycle

To evaluate the proportion of proliferating cells, the cells were fixed, stained with FITC-labeled antibodies to Ki-67 [26], and subjected to flow cytometry analysis. At Figure 4(a)(A, B), one can see the results of FACS for cell populations cultivated in the presence of gDNA and GC-DNA (50 ng/mL, 0.5 and 3 h). gDNA within 30 minutes induces a decrease of the quantity of cells that express Ki-67. As early as 3 hours after adding gDNA, the marker level approaches the reference values. GC-DNA have virtually no effect on the quantity of Ki-67+ cells.

A similar analysis was performed for proliferating cell nuclear antigen (PCNA) [27]. After the cells had been exposed to 50 ng/mL of gDNA and GC-DNA, the level of PCNA protein in the cells increased as compared to control (Figure 4(b)(A, B)). Interestingly, the level of PCNA in the cells increased when cells had been exposed to gDNA, thus, producing overall a picture substantially different from that of Ki-67 staining (Figure 6(a)).

Because the two proliferation markers showed different results regarding the change in the level of cells' proliferative activity after an increase of ecDNA concentration, an additional examination of the expression of three genes involved in the cell cycle regulation was performed.

It is known that cell cycle initiation activates the synthesis of cyclin D1 protein encoded by* CCND1* gene. Cyclin D1 launches G1 phase of the cell cycle and plays a key role in the regulation of cell transfer from G1 to S phase [28]. The expression level of CCND1 gene is regulated at the stage of transcription. In haMSC exposure to gDNA fragments, a slight lowering of the level of* CCND1* gene expression occurs in 20 minutes after adding DNA fragments to the culture medium (Figure 4(c)). GC-DNA induces 1.4-fold raised* CCND1* gene expression in 1 hour after the beginning of exposure. In 24 hours, the* CCND1* gene expression level in haMSC increases on average 1.2–1.5 times higher when exposed to gDNA and pBR322 and 2.2 times higher when exposed to GC-DNA (Figure 4(c)).

We also analyzed changes of expression of two cyclin-dependent kinase inhibitors that participate in the regulation of cell cycle.* CDKN2* encodes p16 protein, which disturbs binding of cyclin D1 to CDK [29, 30]. The other protein, p21, refers to WAF/Kip CDKI family and is encoded by* CDKN1A *gene. The p21 kinase inhibitor negatively affects the activity of cyclin-depended kinase (CDK2) complexes at G1 phase of the cell cycle and cyclin-depended kinase (CDK1) complexes at G2 phase of the cell cycle [28]. Expression of this gene is controlled by p53 protein. Raised* CDKN1A *gene expression can result in G1-arrest.* CDKN2* and* CDKN1A* gene expression in haMSCs was found to be increased 1.6–1.9 times within the first hour of gDNA exposure. GC-DNA and pBR322 did not have statistically significant effect on CDKN2 and CDKN1A gene expression (Figure 4(c)).

The data collection presented at Figure 4 suggests that gDNA, within the first hour after being added to the culture medium of haMSC, temporarily blocks the cell cycle (reducing Ki-67 protein level, lowering amount of mRNA* CCND1*, and raised amounts of mRNA* CDKN2* and* CDKN1A*). GC-DNA, contrariwise, insignificantly induces proliferation.

The discordance of observed changes in the staining for PCNA (Figure 4(b)) and Ki-67 (Figure 4(a)) after exposing to gDNA and GC-DNA could be explained by shifting the nuclear processes toward DNA repair [31, 32]. Importantly, PCNA is involved in both DNA replication and DNA repair. Moreover, PCNA was shown to serve as a biomarker of DNA repair and as processivity factor for DNA polymerase-*δ* that repairs DNA gaps after the excision of damaged DNA strand.

Increased expression of *γ*H2AX histone together with no cell cycle arrest in spite of ROS generation and double-stranded breaks after GC-DNA exposure suggests induction of rapid and effective DNA damage response (DDR) by GC-DNA fragments. Existence of such a response in haMSCs, which causes their higher resistance to DNA damage, was proven earlier [33, 34]. During DDR, expression of genes determining DNA repair and survival of affected genes increases in the cells. We analyzed a possibility of the DDR in cells incubated in the presence of gDNA and GC-DNA in the medium.

### 3.4. Exposure to Either gDNA or GC-DNA Supports Cell Survival

**Figure 5:**
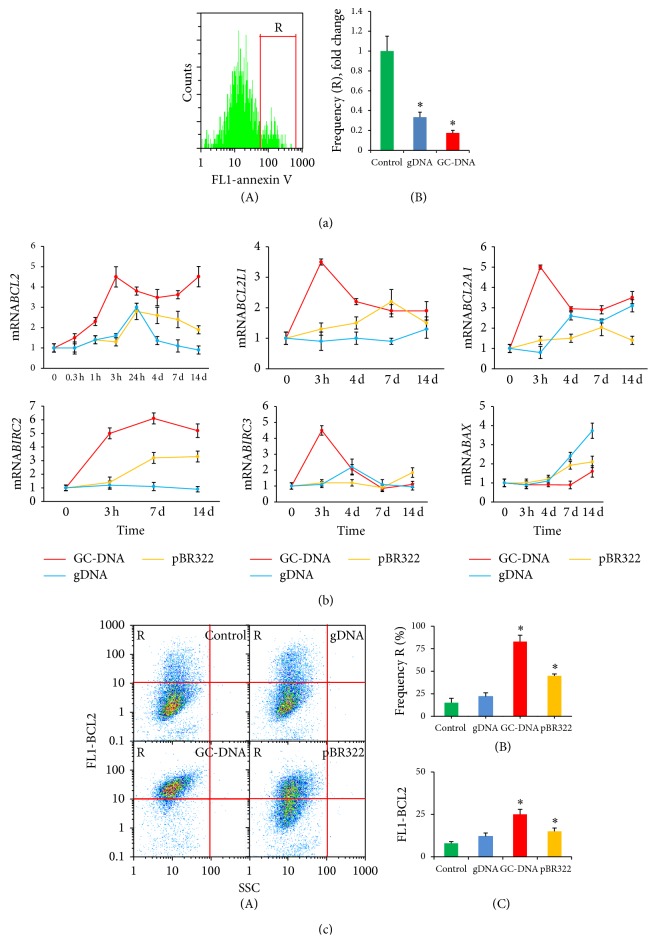
Exposure to either gDNA or GC-DNA supports cell survival. (a) Detection of haMSCs with sings of early apoptosis (FACS). HaMSCs were exposed to DNA samples (50 ng/mL, 3 h). (A) The distribution of fluorescence intensities of the cells stained with Annexin V-FITC; (B) the proportion of Annexin V-positive cells in total cell population. (b) The ratio of the levels of mRNA* BCL2*,* BCL2A1* (Bfl-1/A1),* BCL2L1* (BCL-X),* BIRC3* (c-IAP1), and* BIRC2* in cells exposed to 50 ng/mL gDNA, pBR322, or GC-DNA. The incubation time is indicated on the histogram. (c) Detection of BCL2 protein level in haMSCs exposed to DNA samples (50 ng/mL, 14 days). Cells were fixed with 3% PFA, treated with 0.1% Triton X-100 and stained with anti-BCL2 (FITC) antibodies. (A) FACS analysis showing the stained cellular fractions (SSC-FL1 (BCL2) diagram). Gate R encircles the fraction of haMSCs that express large amounts of protein BCL2; (B) the proportion of cells with large amounts of protein BCL2 (gate R); (C) the average of the median signal intensity of FL1 (BCL2) in haMSCs. Horizontal bars reflect relative expression levels in the control cells. Data points were averaged and represented as mean ± SD for three biological replicates. Asterisk (^*^)  depicts the differences between exposed cells and control cells that were statistically significant by the Mann-Whitney* U* test (*P* < 0.05).

To quantify cells in early apoptosis, we used FITC conjugated Annexin V (Figure 5(a)) and FACS. After three hours of exposure either to gDNA or GC-DNA, the proportion of the apoptotic cells in treated cultures decreased to levels 3 and 5 times less than those in the control haMSCs.

In three hours after adding GC-DNA to haMSC culture medium, levels of mRNA for* BCL2*,* BCL2A1* (Bfl-1/A1),* BCL2L1* (BCL-X),* BIRC3* (c-IAP1), and* BIRC2* increase 3 to 7 times (Figure 5(b)). In case of treatment with gDNA or pBR322, these genes also tend to increase their mRNA biosynthesis, up to 1.5–2.5 times. After long-lasting growth in the presence of DNA probes, increased level of expression of antiapoptotic genes* BCL2*,* BCL2A1*,* BCL2L1*, and* BIRC2 *is retained in the presence of GC-DNA and pBR322, and* BCL2A1 *expression remains elevated in the presence of gDNA.

High level of mRNA for* BCL2* in haMSC after 14-day long incubation with pBR322 or GC-DNA well correlates with an increase in the amount of BCL2 protein itself, as detected using FACS (Figure 5(c)). Both DNA probes induce an increase of the ratio of cells with very high level of expression of this antiapoptotic protein (Figure 5(c)(A)) in the cell pool. The effect of an exposure to GC-DNA is significantly more prominent than that of the action of pBR322 or gDNA (Figure 5(c)).

After long-time cultivation of haMSC in the presence of DNA probes the level of expression of the antiapoptotic proteins declines. However we found an increase of expression of the gene for a proapoptotic protein BAX (Figure 5(b)).

### 3.5. GC-DNA Induces Adipogenic Differentiation in haMSCs

**Figure 6:**
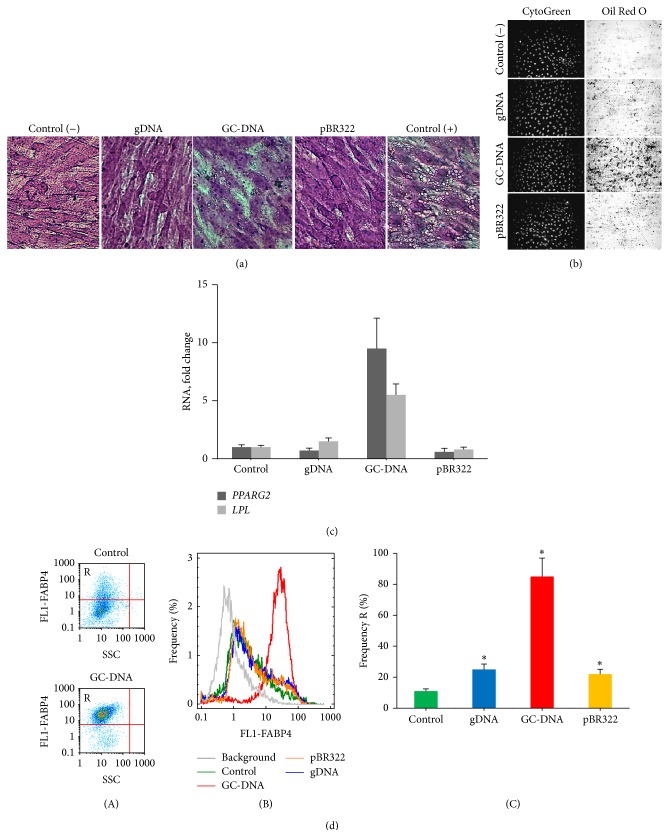
GC-DNA induces adipogenic differentiation of haMSCs. HaMSCs were grown to subconfluency (~80%) and then DNA samples were added. Cells were cultivated in the presence of DNA samples (50 ng/mL) at 37°C for 14 days in AmnioMax Basal Medium with AmnioMax Supplement C100 (Gibco). In a week the culture medium with DNA samples was refreshed. (a) Changes in the morphology of the cultured cells (100x). Cells were fixed with isopropanol and stained with crystal violet. Control (−) DNA samples were not added to the medium; control (+) cells were cultured in the medium reliably inducing adipogenesis (StemCell Technologies Inc.). (b) Adipogenic differentiation of haMSCs was identified by fixing the cells with 4% PFA and staining with 0.3% Oil Red O solution and with CytoGreen (20x). (c) Relative levels of RNA for* PPARG2* and* LPL. *The cells were fixed after 7 days of incubation. (d) Detection of FABP4 protein level in haMSCs exposed to DNA samples. Cells were fixed with 3% PFA, treated with 0.1% Triton X-100 and stained with anti-FABP4 (FITC) antibodies. (A) FACS analysis showing the stained cellular fractions (SSC-FL1 (FABP4) diagram). Gate R encircles the fraction of haMSCs that express large amounts of protein FABP4; (B) the distribution of fluorescence intensities of the cells stained with anti-FABP4 (FITC) antibodies; (C) the proportion of cells with large amounts of protein FABP4 (gate R). Horizontal bars reflect relative expression levels in the control cells. Data points were averaged and represented as mean ± SD for three biological replicates. Asterisk (^*^)  depicts the differences between exposed cells and control cells that were statistically significant by the Mann-Whitney* U* test (*P* < 0.05).

We incubated haMSCs in the presence of 3 different DNA probes (gDNA, GC-DNA, and pBR322) in a concentration of 50 ng/mL during 14 days, using the standard culture medium, with no differentiation-inducing factors added. Within 7 days the culture medium was replaced with second addition of DNA probes in the same concentration. As negative control, cells without DNA probes added were used, and, as positive control, cells were used that had been cultivated in a medium containing standard adipogenic differentiation-inducing factors (StemCell Technologies Inc.). 14 days after the experiment was started, we visually recorded considerable changes in the morphology of cells cultivated in the presence of GC-DNA (Figure 6(a)). Cells containing characteristic small lipoid granules accumulated in the haMSC population. Such cells neighbored other cells, which did not differ morphologically from the controls. In the presence of gDNA and pBR322 probes, cells generally showed no considerable difference from the controls in morphological respect. However, there were also some single cells that included small lipoid granules.

To prove adipogenic differentiation, the cells were stained with Oil Red O (Figure 6(b)). Of 3 DNA samples, the GC-DNA sample only induced formation of a large number of stainable lipoid granules in the haMSC population. We applied additional markers for corroboration of adipogenesis of haMSCs in the presence of GC-DNA. The PPARG transcription factor is known as a differentiation-inducing agent, expression of which increases during the process of adipogenesis [35, 36]. GC-DNA was shown to stimulate a 10-fold increase of* PPARG2* mRNA level in haMSCs (Figure 6(c)) a week after the experiment was started, while gDNA in the same conditions did not induce any elevation of expression of this gene. Along with the elevation of expression of* PPARG2*, the presence of GC-DNA in the medium increased several times the amount of RNA for LPL protein, expression of which increases in preadipocytes and adipocytes [36].

14 days after GC-DNA was added to the culture medium of haMSCs, we recorded a drastic increase of the amount of protein encoded by* FABP4 *gene, in the cells. Fatty acid binding protein (FABP4) accumulation was evaluated using flow cytometry (Figure 6(d)). About 90% of the cells, incubated with GC-DNA, expressed this marker in large amounts (Gate R, Figure 6(d)). In control haMSCs, mere 10% of the cells expressed large amounts of this protein. The gDNA and pBR322 probes stimulated extra (in reference to the control) FABP4 protein synthesis in less number of cells than GC-DNA (about 20% of the whole cell population) (Figure 6(d)(B, C)).

**Figure 7:**
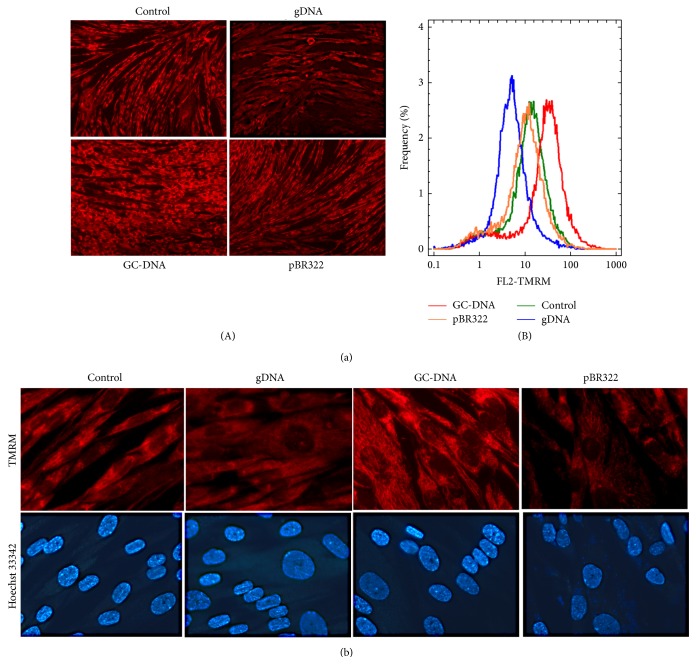
Mitochondrial biogenesis increased with adipogenic differentiation of haMSCs in the presence of GC-DNA. (a) HaMSCs were grown to subconfluency (~80%) and then DNA samples were added. Cells were cultivated in the presence of DNA samples (50 ng/mL) at 37°C for 7 days in AmnioMax Basal Medium with AmnioMax Supplement C100 (Gibco). (A) Cells were stained with MitoTracker TMRM and were photographed at the same exposure (20x). (B) FACS analysis showing the distribution of fluorescence intensities of the cells stained with TMRM. (b) Cells were cultivated in the presence of DNA samples (50 ng/mL) at 37°C for 14 days; in a week the culture medium with DNA samples was refreshed. Cells were stained with MitoTracker TMRM and Hoechst 33342 (100×).

An additional verification of adipogenesis of haMSCs in the presence of GC-DNA is elevation of mitochondrion potential, which was tested with TMRM mitotracker (Figure 7) using fluorescence microscopy and FACS analysis. As found earlier, the weight of MSCs mitochondria considerably increases in the course of adipogenic differentiation [37]. The gDNA and pBR322 probes induced the inverse effect: a reduction of the mitochondrion potential. Interestingly, after 2 and more weeks from the start of haMSCs cultivation, in the presence of all the DNA probes in the medium, the cell population begins to contain large cells with enlarged nucleus and reduced level of staining detected with mitotracker (Figure 7(b)).

Therefore, we have established that the GC-rich human DNA sequence (rDNA) as part of extracellular DNA of the medium induces differentiation of haMSCs to adipocytes, even without the usual adipogenesis-inducing factors.

## 4. Discussion

The main result of our work is the following: GC-rich fragments of human genomic DNA, being added to the culture medium of haMSCs, evoke spontaneous adipogenic differentiation of these cells. The source of GC-rich fragments inducing adipogenesis was a GC-DNA-carrying plasmid, which contained a GC-rich insertion, namely, a fragment of human rDNA, in the pBR322 bacterial vector. The fact of differentiation of haMSCs to adipocytes in the presence of the model GC-DNA is corroborated using several independent standard techniques: morphological alterations of the cells (Figure 6(a)), cell staining with the Oil Red O dye (Figure 6(b)), expansion in the amounts of RNA for PPARG transcription factor and for marker protein LPL (Figure 6(c)), and a marked increase in the content of FAPB4 protein in the cells (Figure 6(d)).

When haMSCs were cultivated in the presence of GC-DNA, we observed other effects, which, according to the published works, accompany or even cause adipogenesis. For example, GC-DNA causes augmentation of the mitochondrion potential (Figure 7). It was earlier demonstrated that increased mitochondrial activity is a prerequisite for the MSC differentiation into adipocytes [37].

Additionally, an exposure to DNA fragments added to the culture medium, irrespective of their GC-content, also induced nuclear DNA breaks (Figure 3). Spontaneous DNA fragmentation was recently observed at the early stage when adipocyte commitment occurs [38]. This DNA damage was independent from either spontaneous or induced apoptosis and probably was part of the differentiation program [38]. DNA breakage at early stages of adipogenic differentiation induced by ecDNA fragments was accompanied by an activation of DNA repair. Three facts point at this kind of cell response. Expression of* BRCA1* gene (Figure 3(d)) [39] and expression of the phosphorylated form of H2AX histone (Figure 3(b)) [19, 39] increase. The quantity of PCNA protein (participates in excision repair as a cofactor of DNA-polymerase delta [31]) increases as well against the background of minor change of the amount of Ki-67, another proliferation marker (Figure 4). Thus, after an exposure to DNA fragments, we observed a development of DNA damage response (DDR). DDR is a program that preserves genome integrity and suppresses malignant transformation. Recent studies in stem cells suggest that cell differentiation is under the influence of DDR program, too [25, 33, 34, 40–44]. GC-DNA induced much more effective DDR than pBR322 or gDNA did. Induced by GC-rich DNA nuclear DNA breaks were repaired much faster than those induced by gDNA (Figure 3), perhaps, due to more considerable rise of expression of the proteins, which participate in the reparation process (BRCA1 (Figure 3(d)) and PCNA (Figure 4(b))).

In the course of cultivation of haMSC together with DNA fragments added to culture medium we observe reduction of the cell apoptosis rate (Figure 5(a)). Recently, the authors of [45] suggested a shift in the balance between proapoptotic and antiapoptotic molecules during adipogenesis resulting in a higher apoptosis resistance. As a cause of higher resistance of haMSC to apoptosis during differentiation, increased expression of BCL2 gene was pointed out [46]. In this study, we likewise detected a significant increase in the amounts of mRNA for* BCL2* and of this protein itself in haMSC, which had been cultivated in the presence of GC-DNA fragments (Figures 5(b) and 5(c)). Except for BCL2, expression of other antiapoptotic genes of the same family and those of BIRC family significantly increased. After long-time cultivation of haMSC cells in the presence of gDNA and pBR322 fragments we observed elevated expression of the BAX proapoptotic gene (Figure 5(b)). This response correlates with the emergence of large cells with damages mitochondria in the cell pool. In case of GC-DNA, this response is insignificant.

We believe that the prime cause of the above changes in haMSC is transient generation of ROS in response to the change in concentration of ecDNA in the culture medium. Many researchers point at the fact that differentiation-inducing agents induce ROS generation. Reactive oxygen species mediate adipocyte differentiation in mesenchymal stem cells [47–49]. It was suggested that the increase in the intracellular ROS level via Nox4 mediates adipocyte differentiation in haMSCs [50]. gDNA, pBR322, and GC-DNA induced elevation of the ROS level in hAMSCs, and GC-DNA appeared to be a stronger inducing factor for ROS generation than gDNA and pBR322. The mechanism of ROS generation stimulation in the presence of added ecDNA fragments is still unknown and requires separate studies. Interestingly, the ROS level in hAMSCs elevates virtually at once after the ecDNA concentration has increased. It can point at a suggestion that the DNA fragments induce ROS production in the place of binding to the cell membrane, seemingly, through an alteration of activity of the NOX family enzymes involved in ROS production. We revealed earlier a similar mechanism when studying the action of DNA fragments upon MCF-7 cancer cells [51].

It should be noted that all the three DNA samples added to the culture medium of haMSC initiated similar responses in the cells (ROS burst, DNA breaks, and antiapoptotic response). But the magnitude of the cell response to treatment with ecDNA was much higher in case of GC-DNA, which, along with bacterial vector, contains a GC-rich fragment of the transcribed region of human rDNA (Figure 1). Thus, biological effect of GC-DNA on haMSC can not be explained solely by high GC-content and existence of sequences that are ligands for TLR9. Vector pBR322 (53% of GC-pairs) contains the same GpC–motifs, which are ligands for TLR9, as the GC-DNA carrying plasmid, but has ill-defined activity in respect with adipogenesis induction at the concentrations used (50 ng/mL). One can speculate that there are specific GC-rich motifs within human rDNA (and other GC-rich sequences of human genome), which determine binding of ecDNA to the cell receptors and ensure penetration of DNA fragments into the cells.

One can suggest a speculation, which requires further experimental testing. As we found earlier, GC-rich regions of human DNA differ from bacterial GC-rich DNA by very high content of Gn sequences [52] and Figure 1. Further, guanosine within the Gn motifs is extremely easily oxidized to 8-oxo-guanosine [18, 53]. It was shown for cancer cells that sequences containing oxidized bases easily penetrate to the cytoplasm regions located close to the nucleus and induce sharp increase in ROS formation [52]. Presumably, high content of easily oxidized Gn as part of rDNA in comparison with pBR322 vector underlays the high biological activity of GC-DNA. gDNA undoubtedly includes Gn as well, but their content is not high because of low total content of GC-pairs, and at low concentration gDNA is added; they exert no marked effect on haMSCs. Existence of ligands within rDNA, which bind to still unidentified DNA-recognizing receptors, like well-known TLR9, should not also be ruled out.

Further studies are required to reveal the molecular machinery that underlays the action of GC-rich DNA as adipogenic differentiation inducer. At the same time, future studies should take into account the fact that, in case of some pathologies characterized by extended apoptosis, the circulating DNA (cell-free DNA and cfDNA) is enriched with a fraction of GC-rich human genome sequences. In particular, the content of rDNA can increase by a factor of 10 and more in case of some diseases, such as rheumatoid arthritis or ischemic heart disease [11–13]. Under these circumstances, own stem cells of the body, as well as haMSCs used for therapy, can differentiate between adipose tissue cells. Adipogenesis of haMSC in the presence of altered, GC-rich cfDNA can be a potential factor of obesity and osteoporosis.
